# Effect of BMAP-28 Antimicrobial Peptides on *Leishmania major* Promastigote and Amastigote Growth: Role of Leishmanolysin in Parasite Survival

**DOI:** 10.1371/journal.pntd.0001141

**Published:** 2011-05-31

**Authors:** Miriam A. Lynn, Jason Kindrachuk, Alexandra K. Marr, Håvard Jenssen, Nelly Panté, Melissa R. Elliott, Scott Napper, Robert E. Hancock, W. Robert McMaster

**Affiliations:** 1 Immunity and Infection Research Centre, Vancouver Coastal Health Research Institute, and the Department of Medical Genetics, Vancouver, Canada; 2 Centre for Microbial Diseases and Immunity Research and the Department of Microbiology and Immunology, University of British Columbia, Vancouver, Canada; 3 Department of Science, Systems and Models, Roskilde University, Roskilde, Denmark; 4 Department of Zoology, University of British Columbia, Vancouver, Canada; 5 Vaccine and Infectious Disease Organization, University of Saskatchewan, Saskatoon, Canada; 6 Department of Biochemistry, University of Saskatchewan, Saskatoon, Canada; Lancaster University, United Kingdom

## Abstract

**Background:**

Protozoan parasites, such as *Leishmania*, still pose an enormous public health problem in many countries throughout the world. Current measures are outdated and have some associated drug resistance, prompting the search into novel therapies. Several innovative approaches are under investigation, including the utilization of host defence peptides (HDPs) as emerging anti-parasitic therapies. HDPs are characterised by their small size, amphipathic nature and cationicity, which induce permeabilization of cell membranes, whilst modulating the immune response of the host. Recently, members of the cathelicidin family of HDPs have demonstrated significant antimicrobial activities against various parasites including *Leishmania*. The cathelicidin bovine myeloid antimicrobial peptide 28 (BMAP-28) has broad antimicrobial activities and confers protection in animal models of bacterial infection or sepsis. We tested the effectiveness of the use of BMAP-28 and two of its isomers the D-amino acid form (D-BMAP-28) and the retro-inverso form (RI-BMAP-28), as anti-leishmanial agents against the promastigote and amastigote intracellular *Leishmania major* lifecycle stages.

**Methodology/Principal Findings:**

An MTS viability assay was utilized to show the potent antiparasitic activity of BMAP-28 and its protease resistant isomers against *L. major* promastigotes *in vitro*. Cell membrane permeability assays, caspase 3/7, Tunel assays and morphologic studies suggested that this was a late stage apoptotic cell death with early osmotic cell lysis caused by the antimicrobial peptides. Furthermore, BMAP-28 and its isomers demonstrated anti-leishmanial activities against intracellular amastigotes within a macrophage infection model.

**Conclusions/Significance:**

Interestingly, D-BMAP-28 appears to be the most potent antiparasitic of the three isomers against wild type *L. major* promastigotes and amastigotes. These exciting results suggest that BMAP-28 and its protease resistant isomers have significant therapeutic potential as novel anti-leishmanials.

## Author Summary

Protozoan parasites are the causative agent of much disease in tropical areas of the world. Currently, the control of these diseases is dependent on outdated drug treatment, with associated high toxicity and drug resistance. There is an urgent need for novel anti-parasitic therapies. One emerging anti-parasitic therapies is Host defence peptides (HDPs). Here we test the HDP BMAP-28 as an anti-leishmanial therapy against two lifecycle stages of *Leishmania major*, the promastigotes (insect infective form) and the intracellular amastigote (mammalian infective form). Two stereoisomers of BMAP-28, the D-amino acid form (D-BMAP-28) and the retro-inverso form (RI-BMAP-28), were also tested for anti-leishmanial activity. The BMAP-28 form (L-form) was susceptible to degradation by GP63, the metalloproteinase that covers the promastigotes cell surface. However, the BMAP-28 isomers, the D-form and RI-form were resistant, and therefore more potent against the promastigote parasite. Though other anti-leishmanial HDP studies focus on the promastigote form of the parasite, it is the mammalian infective form, the amastigote, which causes the disease symptoms. Here we demonstrate that BMAP-28 and its isomers D-BMAP-28 and RI-BMAP-28 are effective against the amastigote form of the parasite using a macrophage infection model. These findings show that BMAP-28 has excellent potential as a novel anti-leishmanial therapeutic.

## Introduction

The protozoan parasite *Leishmania*, the causative agent of leishmaniasis, represents a global health problem that is prevalent in Europe, Africa, Asia and the Americas. Up to 20 million people are affected [1]. Leishmaniasis presents one of three distinct categories of diseases; visceral, cutaneous and mucocutaneous. Visceral leishmaniasis is the most severe form of the disease; it causes a chronic disseminating disease of the liver and spleen, and can be fatal if left untreated [2]. The cutaneous disease generally presents as self-healing cutaneous lesions; however, with certain species can subsequently metastasize resulting in debilitating mucocutaneous disease [2].

*Leishmania* have a digenetic lifecycle consisting of the morphologically distinct promastigote and amastigote stages. The motile flagellated stage, the promastigote form, reside extracellularly in sand fly vectors of the genera *Phlebotomus* or *Lutzomyia* [3]. Virulent metacyclic promastigotes are inoculated into the dermis of mammalian hosts during sand fly blood meals. Macrophages rapidly internalize the promastigotes into a parasitophorous vacuole, which fuses with lysosomes to generate an acidic compartment containing hydrolytic enzymes. This triggers differentiation into the aflagellated obligate intracellular stage, the amastigote, which initates the onset of disease symptoms in the host [4].

Traditional drug treatments consisit of polyvalent antimonial therapy; repeated infections of the human population by either zoonotic or anthroponotic cycles coupled with ineffective treatments have led to selection of antimony-resistant parasites that may cycle through the human reservoirs leading to increase drug resistance within populations [5]. Furthermore, the efficacy of leishmaniasis therapeutics has also been threatened by the growing incidence of multidrug resistance [6], [7], emphasizing the need for novel therapies. In the search for novel anti-infective therapies host defence peptides (HDPs), evolutionarily conserved defence molecules found in all living species, are an intriguing alternative.

HDPs are central components of the defence mechanisms of organisms ranging from bacteria to animals. These peptides are defined by their amphipathic nature, cationicity and relatively small size. The cationicity and hydrophobic nature of HDPs promote their interaction with cell membranes and in particular the negatively charged membranes of Gram-positive and Gram-negative bacteria. Although the antimicrobial activity of HDPs was initially attributed directly to cell membrane lysis, HDPs can also translocate across cell membranes and interact with various internal microbial targets such as DNA [8], [9] and DnaK [10], [11]. Indeed, the translocation of HDPs across eukaryotic cell membranes and subsequent interaction with cell vacuoles has also been demonstrated [12]. Thus, in addition to their direct antimicrobial activities, there is an emerging appreciation for the ability of HDPs to modulate host immune responses. These immunomodulatory activities include the up-regulation of cytokines/chemokines and their receptors, leukocytes recruitment, stimulation of histamine release from mast cells, angiogenesis, dendritic cell maturation, wound healing, and the ability to modulate the potentially pathological inflammation that can be associated with bacterial infection (sepsis) [13], [14], [15], [16], [17], [18]. These HDP activities include anti-protozoan and anti-leishmanial activities [12], [19], [20], [21], [22]. Furthermore, the pleotropic activities of HDPs make them compelling templates for the development of novel anti-infective therapeutics.

Recently, bovine myeloid antimicrobial peptide 28 (BMAP-28), a cathelicidin found in bovine neutrophils, has been demonstrated to confer protection to bacterial infection and sepsis in animals [23], [24]. BMAP-28 has also been demonstrated to initiate the depolarization of mitochondrial membranes via the induction of the mitochondrial permeability transition pore [25].

The therapeutic potential of BMAP-28 may be limited based on its *in vitro* toxicity. However, our recent data has demonstrated that the D- amino acid enantiomer of BMAP-28 and retro-inverso (RI)-BMAP-28, in which all L- amino acids of the parent peptide are replaced by D- amino acids along with reversal of the order of the peptide sequence, may remove these limitations [26]. Previous investigations of various antimicrobial peptides have also demonstrated that enantiomeric replacements significantly reduced hemolytic activity without affecting antimicrobial activity [26], [27], [28], [29]. Thus, BMAP-28 and its protease resistant isomers are intriguing candidates for testing of therapeutic potential against *Leishmania major*.

In this study, L-BMAP-28, D-BMAP-28 and RI-BMAP-28 were shown to be effective therapeutics against *Leishmania* promastigotes *in vitro* with the D- isoform being most effective in reducing promastigote viability. Cell membrane integrity was disrupted upon BMAP-28 interaction, inducing a late stage apoptotic cell death response. Interestingly, the BMAP-28 peptides were also effective at reducing amastigote cell viability within a macrophage infection model. This data convincingly demonstrates that BMAP-28 isoforms D- and RI-BMAP-28 are promising candidates for anti-leishmanial therapies.

## Materials and Methods

### Ethics Statement

All subjects who participated in this study provided informed consent in writing according to the University of British Columbia Clinical Research Ethics Board guidelines and approval.

### Cell lines

*Leishmania major* strains Friedlin (MHOM/IL/80/Friedlin) and Seidman (NHOM/SN/74/Seidman, denoted as *wt*), its leishmanolysin knockout derivative (denoted as *ko*) and the back leishmanolysin transfected line (denoted *ko+*) [20], [30] were used in these studies. These cells were routinely cultured at 26°C in M-199 medium (Gibco, Grand Island, NY), supplemented with 10% heat inactivated fetal calf serum (Hyclone, Logan) and Pen/Strep (Gibco, Grand Island, NY).

THP-1 cells were routinely cultured with DMEM, supplemented with 10% heat inactivated fetal calf serum (Hyclone, Logan), and 2 mM L-glutamine and Pen/Strep (Gibco, Grand Island, NY).

### Peptide synthesis

BMAP-28 (GGLRSLGRKILRAWKKYGPIIVPIIRIG) isomers were chemically synthesized on a Pioneer solid-phase peptide synthesizer (PerSeptive Biosystems, Foster City, CA) using Fmoc (9-fluorenylmethoxy carbonyl) chemistry. The peptide chains were synthesized from the carboxyl terminus to the amino terminus onto [5-(4-Fmoc-aminomethyl-3,5-dimethyloxyphenoxy) valeric acid]-polyethylene glycol-polystyrene (PAL-PEG-PS) resin. Fmoc-protecting groups at the amino terminus were deprotected with piperidine. The peptides were cleaved from the resin with concurrent deprotection of the side chain-protecting groups by treating the resin-bound peptide with trifluoroacetic acid (TFA) (9.3 parts) in the presence of scavengers (anisole-ethyl-methyl sulfide-1,2-ethanedithiol [3∶3∶1]), for 7 hours. The crude peptides were filtered from the resin, and the TFA was evaporated. Diethyl ether was added to the residues to precipitate the crude peptide. The peptides were isolated and purified by high-performance liquid chromatography (HPLC) on Vydac protein C_4_ columns (1.0 by 25 cm) eluting with a linear gradient of 10% buffer A (H_2_O-0.1% TFA)-90% buffer B (acetonitrile-H_2_O [90/10]-0.01% TFA) for 40 min at a flow rate of 3 ml/minute. Fractions of greater than 95% purity were used for the investigation. The purity and molecular weight of the respective peptides were confirmed by matrix-assisted laser desorption ionization (MALDI)-time of flight mass spectrometry on a PE Biosystems Voyager system 4068 (National Research Council, Plant Biotechnology Institute, Saskatoon, Canada) and by amino acid analysis.

### Peptide preparation

The BMAP-28 peptides were reconstituted in sterile H_2_O (Sigma) at 1 mM concentrations. Working stocks were generated from this stock at 0.5 µM and 2 µM concentrations in an appropriate medium.

### Preparation of MTS solutions

A solution of {3-(4,5-dimethylthiazol-2-yl)-5-(3-carboxymethoxyphenyl)-2-(4-sulfophenyl}-2H-tetrazolium, inner salt or MTS (Promega, Madison, WI, USA) was prepared (2 mg/ml in phosphate-buffered saline 0.02 M, pH 7.2, PBS) and stored at −20°C. Phenazine methosulphate (PMS, 0.92 mg/ml) (Sigma-Aldrich, St. Louis, MI, USA) was similarly prepared in PBS (0.02 M, pH 7.2) and stored at −20°C. Both solutions were stored in the dark and combined just prior to use.

### MTS assay

Mid-log phase promastigotes at concentrations of 5×10^6^ per 100 µl/well were seeded in 96-well tissue culture plates. Peptides were added to the cells in volumes of 20 µl. Heat-killed parasites, prepared by incubation at 99°C for 30 minutes, served as negative controls for the MTS assay. Peptides (0.5 or 2 µM) were incubated with the cells for 4 hours. MTS/PMS was combined 5∶1, and 20 µl was added to each well and incubated at 37°C for 3 hours. Absorbances at 490 nm were measured using a plate reader (Versamax, Molecular devices, US).

### Electron microscopy

Following HDP incubation for 4 hours, promastigote parasites were washed twice in PBS, fixed in 5% (w/v) glutaraldehyde in PBS, included incubated with 2.5% (w/v) OsO_4_ for 1 hour, gradually dehydrated in ethanol (30, 50, 70, 90, and 100% [v/v]; 30 min each) and propylene oxide (1 hour), embedded in Epon 812 resin, and observed using a Hitachi H7600 transmission electron microscope (Hitachi, Tokyo, Japan).

### SYTOX assay

The SYTOX green assay for cell membrane permeability has been previously described [21]. Briefly, 10^7^ mid-log phase parasites were washed twice in PBS then incubated in the dark with 1 µM SYTOX Green (Promega) in PBS for 15 minutes. Fluorescence was measured every 5 minutes after peptide addition for up to 2 hours in a microplate reader, with excitation and emission wavelengths of 485 and 520 nm, respectively. The maximum fluorescence control was demonstrated by the addition of 0.5% Triton X-100.

### Caspase assay

5×10^6^ mid-log phase *Leishmania* cells were incubated with 2 µM D-BMAP-28 or 16 µM staurosporine (Sigma-Aldrich, Oakville, Ontario) for various time intervals at 26°C. Caspase 3/7 protease activity was assayed using the Apo-ONE Homogeneous Caspase-3/7 Assay kit (Promega).

### TUNEL assay

5×10^6^ mid-log phase *Leishmania* cells were incubated with 2 µM D-BMAP-28 for various time intervals at 26°C. *Leishmania* cells were treated with Staurosporine (16 µM; Sigma-Aldrich) for 24 hr at 26°. Quantitation of double stranded DNA breaks was assayed using the HT TiterTACS Assay Kit (Trevigen, Gaithersburg, MD). The following modifications to the manufacturer's instructions were made: all centrifugation steps were done at 7840×g for 10 min each, fixation was done in 4% Paraformaldehyde for 10 min and DNase was incubated with cell lysate for 60 min at 37°C to generate the positive control. A total cell number of 5×10^6^ was used for each labeling reaction. The labeling reaction was done in individual centrifugation tubes.

### Murine macrophage cell viability assay

THP-1 cells were seeded into 100 µl volumes at 1×10 5 cells/ml. Cell were incubated in the presence of 5 or 20 µM of L-BMAP, Ri-BMAP and D-BMAP to a final concentration of 1 or 5 µM for 24 or 72 hours respectively. The THP-1 cell viability was assessed by the addition of trypan blue (Sigma aldrich) on a hemacytometer (Table S1) [31].

### Amastigote assay

Peritoneal macrophages were isolated from 4 to 6 week old BALB/c mice by peritoneal lavage in accordance UBC animal care committee. After isolation, macrophages were washed once with 5 ml complete RPMI medium (Gibco) containing 100 units/ml Penicillin/Streptomycin and resuspended in RPMI medium supplemented with 10% fetal bovine serum (FBS) (Hyclone, Logan, USA). 2×10^5^ peritoneal macrophages were seeded into wells of a 24 well plate (Falcon) in a 500 µl volume of complete RPMI medium and allowed to adhere to the coverslips overnight at 34°C in 5% CO_2_. Infections were performed routinely at a ratio of 10 parasites per macrophage. Briefly, macrophages were infected with day 4 stationary phase promastigotes for 4 hours, washed 3 times with RPMI medium, and subsequently cultured in complete RPMI medium for 48 hours. Cells were incubated with 200 µl of 0.5 or 2 µM concentrations of L-, RI- or D-BMAP-28 for 24 hours. Subsequently, macrophages were fixed by incubation in 10% formaldehyde/PBS at room temperature for 10 minutes, permeabilised in 100% methanol for 20 minutes and stained with ProLong Gold antifade reagent with DAPI (Invitrogen, Eugene, USA). The slides were viewed under UV light with an upright fluorescent microscope (Zeiss, Oberkochen, Germany). Images were collected from the microscope with Digital Focus software and parasite burden per macrophage was quantified as an average of 100 macrophages, and expressed as a percentage of the control infection.

### Immunomodulatory activities of L-, D- and RI-BMAP-28

Human venous blood (100 ml) was collected from healthy volunteers in Vacutainer collection tubes containing heparin (Cat No. 362753, BD, Franklin Lakes, NJ). Blood was mixed at a 1∶1 ratio with RPMI 1640 medium (supplemented with 10% (v/v) FBS, 2 mM L-glutamine, and 1 mM sodium pyruvate) and the peripheral blood mononuclear cells (PBMC) were separated by centrifugation in Ficoll-Paque™ PLUS (Cat No. 17-1440-02, GE Healthcare). Peripheral blood mononuclear cells (PBMCs) were isolated from the buffy coat, washed twice in sterile PBS and the numbers of live cells were determined by trypan blue exclusion. PBMCs (0.1 ml at 1×10^6^ cells/ml) were seeded into 96-well tissue culture dishes (Sarstedt), incubated at 37°C in 5% CO_2_ in air and rested for 2 hours before experimental treatment.

To measure cytokine stimulation, PBMCs were treated for 24 hours with L-, D-, and RI-BMAP-28. Monocyte chemotactic protein-1 (MCP-1) was measured in culture supernatants using ELISAs (see below). To test for anti-endotoxin activity, PBMCs were pretreated with 5 µg/ml of HDP for 45 minutes prior to the addition of 10 ng/ml of purified *Pseudomonas aeruginosa* LPS for 4 hours. *P. aeruginosa* LPS was purified as described previously [32]. After 4 hours, secreted TNF-α was measured by ELISA (see below).

Following peptide stimulation for 24 hours, the tissue culture supernatants were centrifuged at 16,000× g (13,000 rpm) at 4°C for 5 minutes in an IEC MicroMax centrifuge to obtain cell-free samples. Supernatants were aliquoted and then stored at −20°C. Secretion of MCP-1 into the tissue culture supernatants was detected by sandwich ELISA (BioSource International and eBiosciences, respectively) (Figure S1). All assays were performed in triplicate. MCP-1 concentration in the culture medium was quantified by establishing a standard curve with serial dilutions of the recombinant human MCP-1. Secretion of TNF-α was monitored in rested PMBCs and cells exposed to peptide and/or LPS (*P. aeruginosa*) by capture ELISA after 24 hrs (eBiosciences).

## Results

### BMAP-28 effects *L. major* promastigote cell viability in a concentration dependent manner

**Figure 1:**
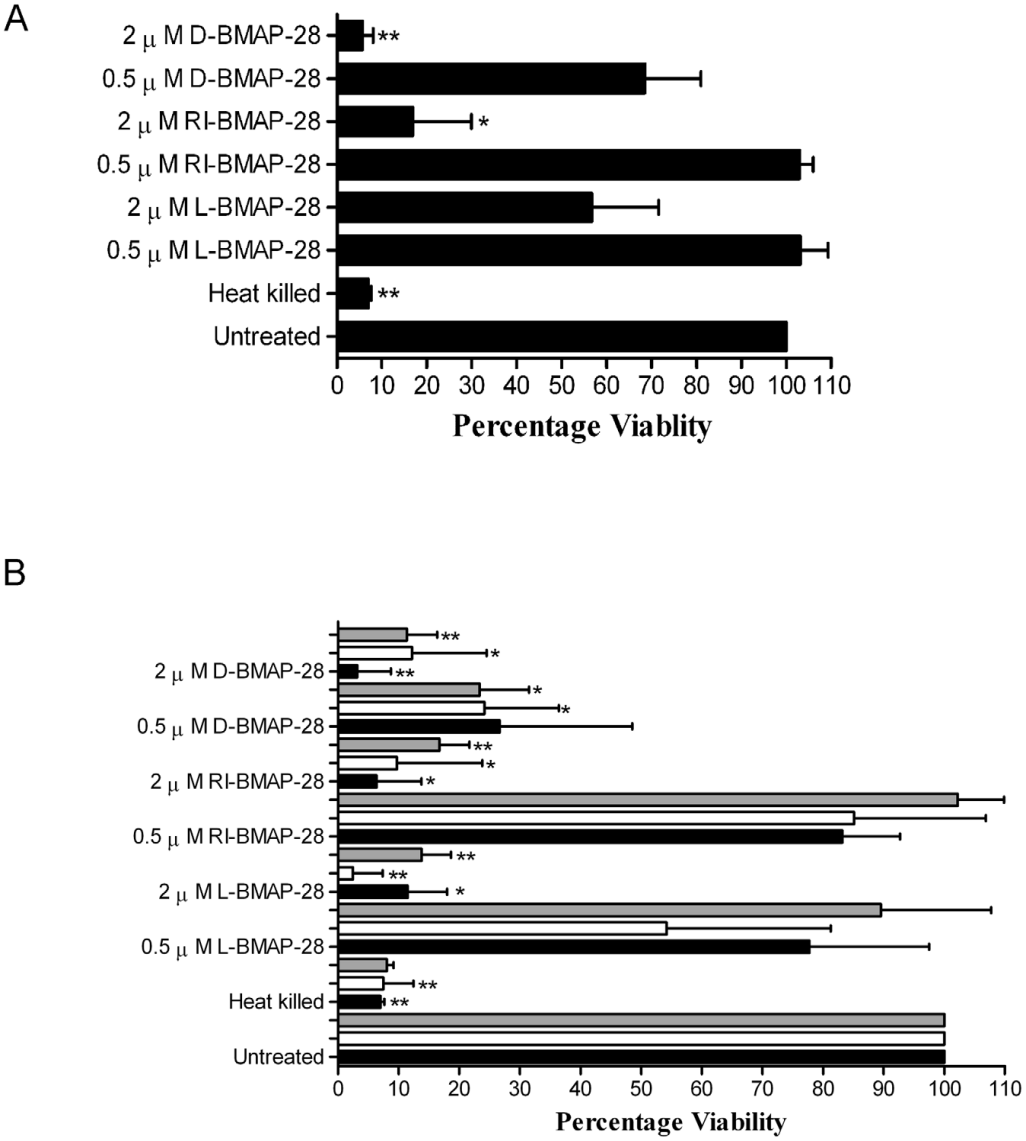
MTS viability assay of *Leishmania major* strains when treated with BMAP-28 variants. (A) *L. major* Friedlin strain was treated with 0.5 µM and 2 µM concentrations of L-, RI- and D-BMAP-28 for 4 hours. (B) *L. major* Seidman strain *wild-type* (*wt*, filled bars), *GP63 knockout* (*ko*, clear bars) and reconstituted (*ko+*, grey bars) were treated with 0.5 µM and 2 µM concentrations of L-, RI- and D-BMAP-28 for 4 hours. Cell viability was assessed at 490 nm and expressed as a percentage of untreated control cells. Three complete biological replicates were performed and the standard errors are shown. Paired T-tests untreated vs treated indicated significance, where *p<0.05, **p<0.005, ***p<0.0005. The bar graph was created using GraphPad prism 4.

To test the anti-leishmanial activities of the BMAP-28 variants, specifically the effects on the viability of promastigote *L. major* Friedlin, an MTS viability assay was performed. An MTS assay measures the mitochondial dehydrogenase levels of viable cells. Unmodified peptide (L-BMAP-28), the retro-inverso isoform (RI-BMAP-28) and the D- amino acid isoform (D-BMAP-28) were incubated with *L. major* Friedlin promastigotes *in vitro* (Figure 1a). Cell viability was assessed at 490 nm and expressed as a percentage of the untreated control. Cells remained 100% viable upon treatment with 0.5 µM L- and RI-BMAP-28, but experienced a 30% reduction in cell viability when treated with D-BMAP-28. Upon addition of 2 µM concentrations of the HDPs, the HDPs demonstrated a concentration dependent effect on *L. major* Friedlin. Cell viability was reduced to 60%, 20% and 5% when treated with L-, RI- and D-BMAP-28, respectively. Of the three BMAP-28 variant forms, the D- form of the peptide was the most potent.

### The effect of protease resistant L-, RI- and D-BMAP-28 on leishmanolysin *ko Leishmania* promastigotes

Previously, the surface metalloprotease leishmanolysin was demonstrated to bestow increased resistance to the antimicrobial activities of HDPs in *L.* major [21]. As RI-BMAP-28 and D-BMAP-28 retained leishmanicidal activity and would be hypothesized to be leishmanolysin resistant we tested the antiparasitic activity of all three BMAP-28 isomers against a leishmanolysin *ko* strain of *L. major* Seidman strain, which lacks the GP63 surface metalloprotease. *L. major* Seidman *wild-type* (*wt*), *L. major ko* and *L. major ko+* (reconstituted version of the Seidman strain, which confers the wild-type phenotype [20], [30]) were incubated with 0.5 and 2 µM L-, RI- and D-BMAP-28 for 4 hours and cell viability was assessed using an MTS viability assay [33].

At both 0.5 and 2 µM concentrations, D-BMAP-28 was the most effective of the three peptides at reducing the cell viability of the *L. major wt* strain (Figure 1b). Though both RI- and D-BMAP-28 remained at similar levels in wild-type, the L- variant was more effective against *L. major ko* than *wt* strains (Figure 1b). Upon re-introduction of the protease to the cells, *L. major ko+*, cell viability levels reverted back to levels similar to wild-type cells; suggesting that in absence of the GP63 protease the L- form of the peptide was more effective at reducing the cell viability, as has been demonstrated for other cathelicidins [20]. However, the effects of the RI- and D- isomers of BMAP-28 on cell viability did not change between *L. major wt* and *ko* strains, demonstrating that they are unaffected by the proteolytic activities of leishmanolysin.

### BMAP-28 analogues induce morphological changes in *L. major*

**Figure 2:**
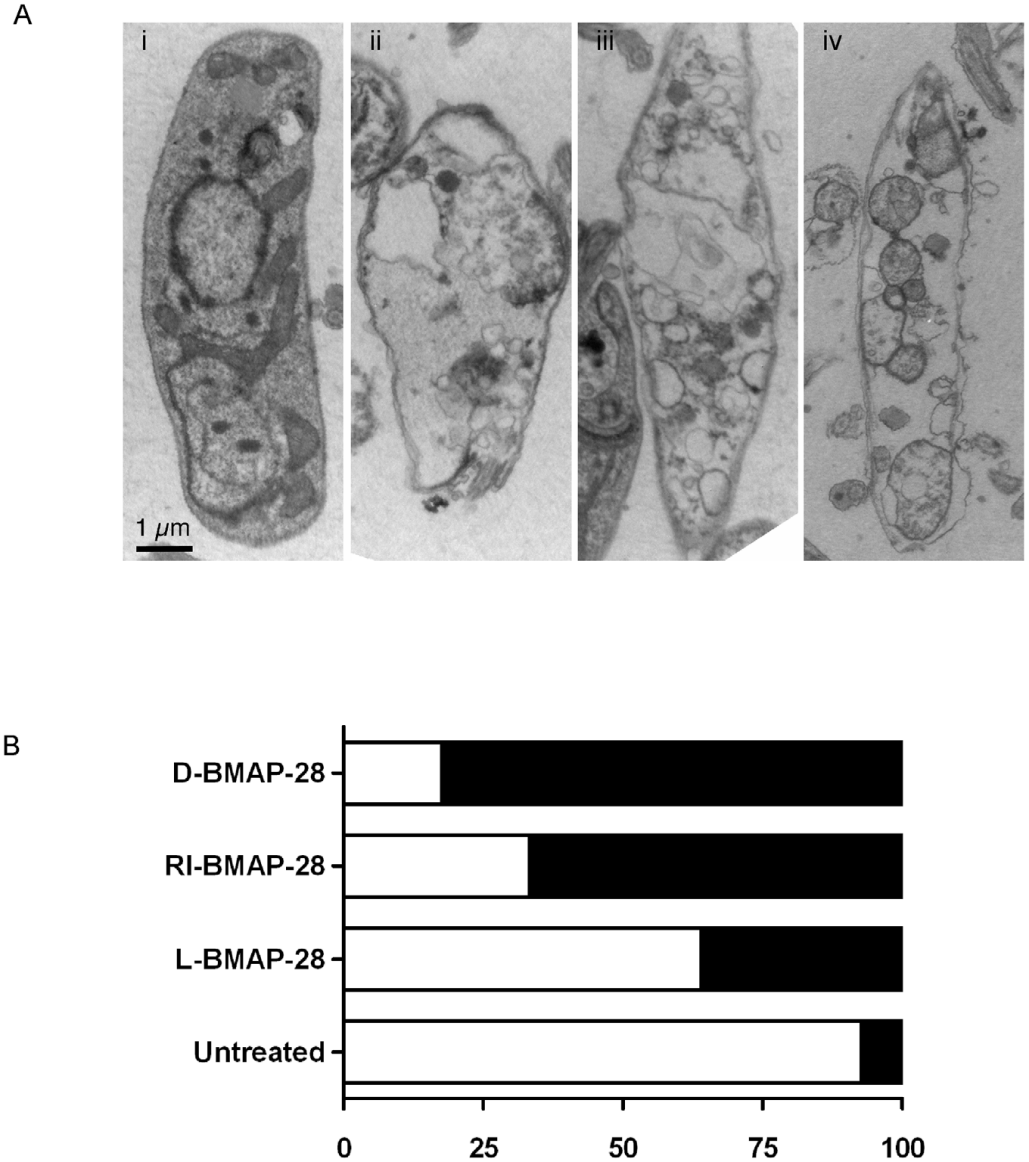
Analyses of effect of BMAP-28 peptides on *L. major* cells. (A) *Wild-type* cells were (i) untreated or treated with (ii) L-BMAP-28, (iii) RI-BMAP-28 or (iv) D-BMAP-28 at 0.5 µM concentrations for 4 hours prior to fixation for TEM analyses. The figure shows cross-sections of cells at ×20,000. Notice that the untreated cell has an intact membrane, an intact cytosol and a few small vacuoles. The treated cells however possess disrupted membranes, large fluid filled vacuoles and loss of cytosolic contents; indicative of osmotic lysis. (B) Quantitative analyses of intact (clear) versus damaged (filled) cells were carried out on *wt* cells in presence/absence of treatment to assess the extent of the damage. Damage was assessed based on three criterion, membrane disruption, loss of cytosolic contents and presence of large fluid filled vacuoles, 120 cells were analysed by TEM for each condition.

To assess morphological changes induced by the BMAP-28 isomers, *L. major* wild-type promastigotes were incubated with 0.5 µM L-, RI- and D-BMAP-28 peptides and prepared for transmission electron microscopy analysis (Figure 2a, b). Cell morphologies were examined based on criterion predictive of osmotic cell lysis: disrupted membrane, loss of cytosol and presence of numerous large empty vacuoles. The results suggested that cells underwent an osmotic cell lysis and followed a similar trend to the MTS viability assay. Cell morphologies were less severe effected in L-BMAP-28 treated cells, and increasingly severe in RI- and D-BMAP-28 treated cells. B-MAP-28 treated cells showed significant cell membrane disruption, losses to cytoplasmic intensity as compared to the untreated cells and significant swelling of cytoplasmic vacuoles (see also Figure S2). This severe morphological change was likely due to osmotic cell lysis and was similar to that seen for other HDPs such as histatin-5 [12].

### BMAP-28 triggers a late apoptotic caspase-independent mechanism of action

Recently, Kulkarni *et al.*, have demonstrated that the HDPs invoke a programmed cell death response in *Leishmania* [21]. They have demonstrated that protegrin, a HDP of the cathelicidin class, induced a non-apoptotic response, whilst pexiganan, a HDP of the magainin class, induced an apoptotic mechanism of cell death [21]. They utilized a SYTOX assay, annexin V staining, and a caspase 3/7 assay in addition to Tunnel assays to determine whether apoptosis was initiated. In this investigation we also sought to determine whether BMAP-28 and its enantiomeric and RI- analogues also followed a similar mechanism of anti-leishmanial activity, to this end we carried out a SYTOX assay, caspase 3/7 assay and a Tunel assay.

**Figure 3:**
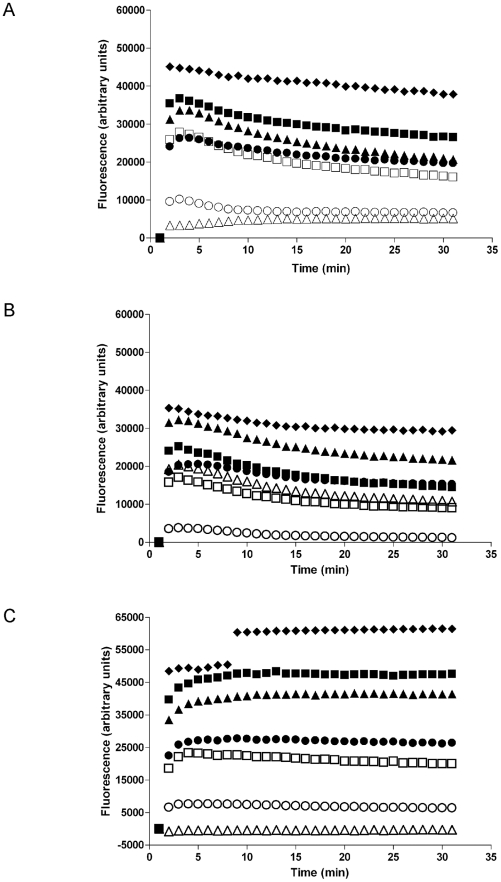
*Leishmania* cell membrane permeabilization by L-, D- and RI-BMAP-28. *L. major* cell lines were treated with 0.5 µM (clear symbols) or 2.0 µM (filled symbols) of each of the three BMAP-28 isomers, L-BMAP-28 (▴, △), RI-BMAP-28 (○, •) and D-BMAP-28 (□, ▪) and analyzed for membrane permeabilization using SYTOX. (A) *L. major wt*, (B) *L. major ko*, (C) *L. major ko+*. Incubation of cells with 0.5% Triton X-100 (filled diamonds) were used as a positive control. Three complete biological replicates were performed and the average is shown, with standard deviations less than 5% for all values.

To examine cell membrane permeability we utilized a SYTOX assay, whereby a SYTOX dye enters cells following membrane disruption and fluoresces upon interaction with DNA. Wild-type, *ko* and *ko+* leishmanial lines were incubated with 0.5 µM and 2.0 µM of L-, D- and RI-BMAP-28 for 150 minutes in the presence of SYTOX (Figure 3). All three cell lines treated with 0.5% Triton X-100 had an immediate rapid and sustained influx of SYTOX. All BMAP-28 analogues demonstrated rapid membrane permeabilization of the *wt* cell line at 2 µM; however, similar permeabilization at 0.5 µM was retained only for D-BMAP-28. In contrast, removal of the GP63 protease (*ko* cell line) resulted in the highest fluorescence of the SYTOX dye, following incubation of the cells with 2 µM L-BMAP-28 (Figure 3b). Reconstitution of GP63 (*ko+*) however, resulted in HDP-mediated membrane permeabilization phenotype similar to those found for the *wt* cells (Figure 3c).

**Figure 4:**
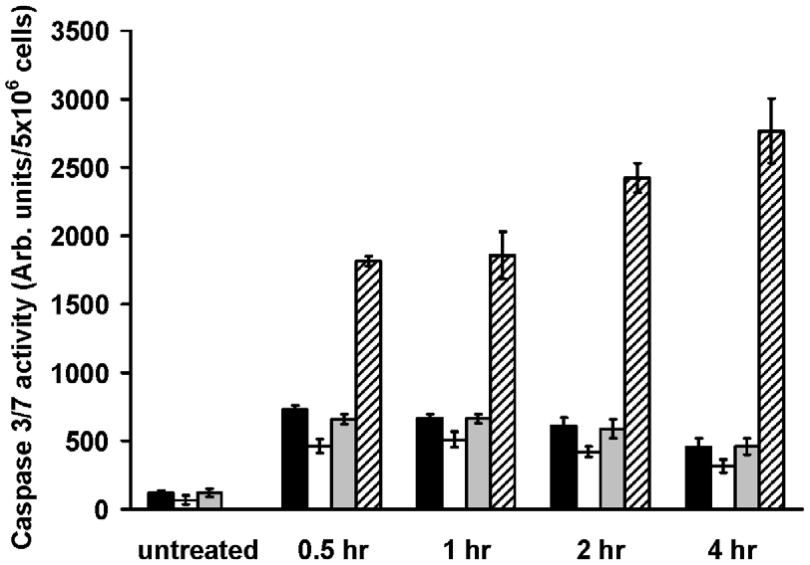
Induction of early apoptosis events in *L. major* strains after incubation with D-BMAP-28. *L. major wt* (black bars), *L. major ko* (clear bars) and *L. major ko*+ (grey bars) were treated with 2 µM D-BMAP-28 for 0.5 hr, 1 hr, 2 hr, and 4 hr and then analyzed for caspase 3/7 activation. *L. major wt* cells treated with 16 µM staurosporine for the indicated period of time were used as positive control (striped bars). Each bar represents the mean of four replicates; error bar represents the standard error of the mean.

Subsequently, we investigated the caspase activity using a caspase 3/7 assay. *L. major wt*, *ko* and *ko+* cells were incubated with 2 µM D-BMAP-28 for various time intervals (<4 hr) and analyzed for caspase 3/7 activation (Figure 4). All three cell lines had minimal levels of caspase 3/7 activation in comparison to the induced caspase dependent apoptotic control, staurosporine. Of these minimal caspase 3/7 activation levels, *L. major ko* consistently had the lowest level compared to *wt* and *ko+*. From these results it was clear that the early D-BMAP-28 induced a caspase independent cell death.

**Figure 5:**
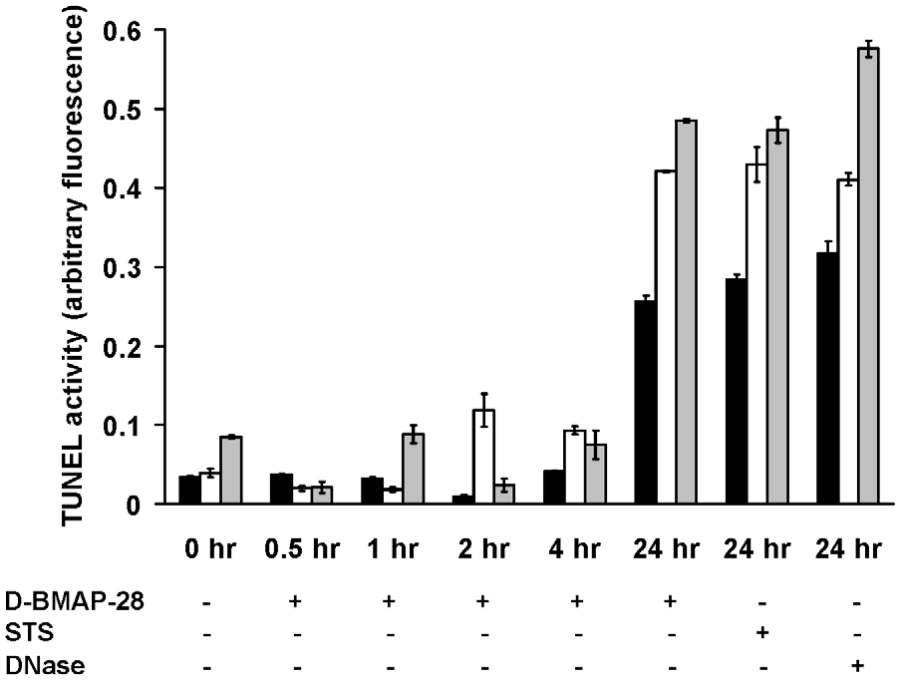
Induction of late apoptosis events in *L. major* strains after incubation with D-BMAP-28. Quantitative colorimetric analysis of DNA degradation in *L. major wt* (black bars), *L. major ko* (clear bars) and *L. major ko*+ (grey bars) after treatment with 2 µM D-BMAP-28 for 0.5 hr, 1 hr, 2 hr, 4 hr and 24 hr using the TUNEL assay. Cells treated with 16 µM staurosporine (STS) as well as DNase treated cell lysates were used as positive controls. Each bar represents the mean of three replicates; error bar represents the standard error of the mean.

Finally, we utilised a Tunel assay to investigate another aspect of apoptosis, DNA degradation in the form of DNA laddering. *L. major wt*, *ko* and *ko*+ cells were treated with 2 µM D-BMAP-28 for time intervals <4 hrs and at 24 hr using the TUNEL assay (Figure 5). Cells were consistently non-apoptotic at time intervals <4 hrs, however, upon 24 hrs incubation DNA laddering was detected and cells designated as apoptotic. D-BMAP-28 produced similar readouts to both apoptotic controls; staurosporine and DNase at 24 hrs. Although maximum readouts differed between the three strains, were levels were consistently higher in *ko* and *ko+* cells.

### *L. major* amastigotes are killed by BMAP-28 isomers in a concentration dependent manner

**Figure 6:**
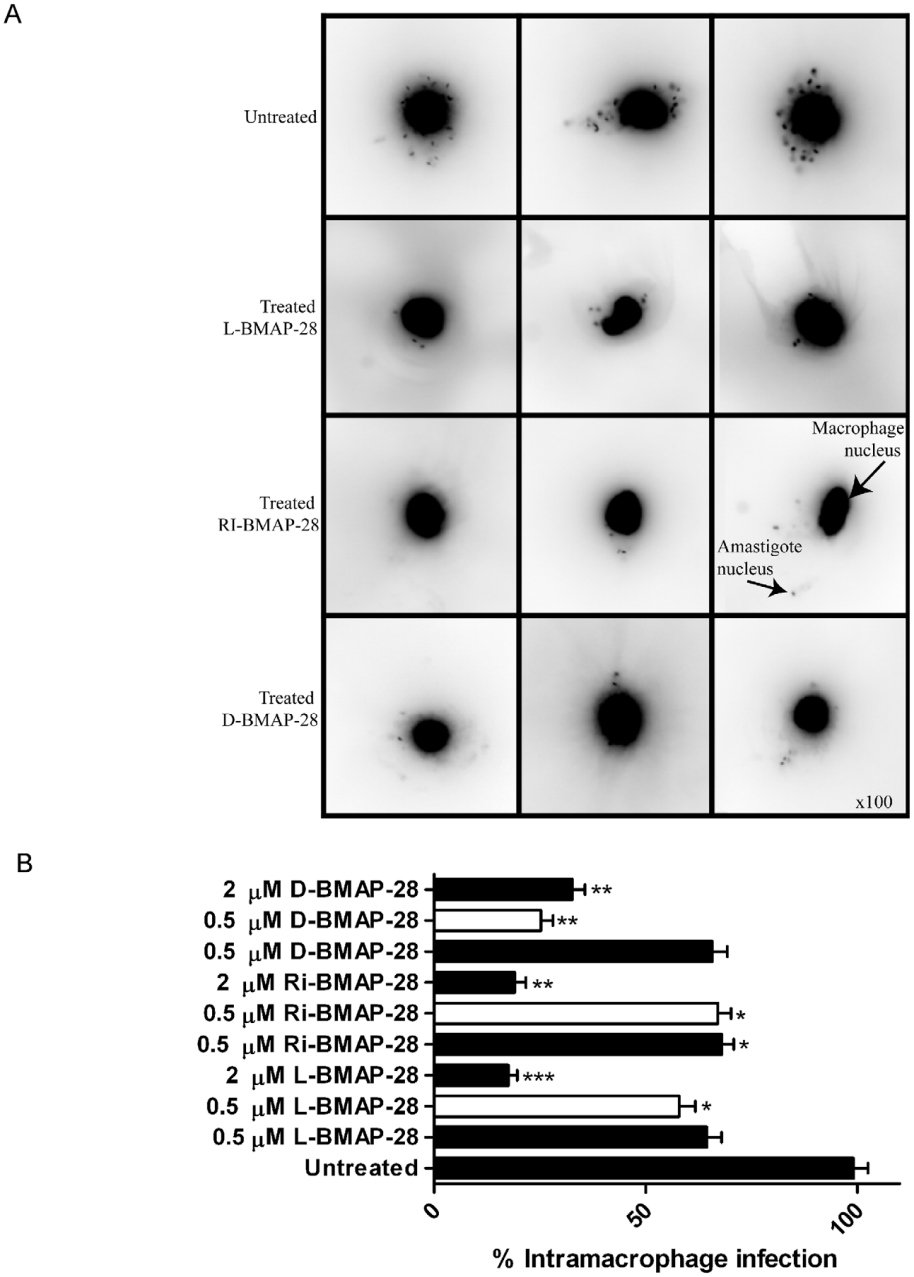
The effect of BMAP-28 isomers on intramacrophage *L. major* infection. (A) The figure displays images that were collected from peritoneal macrophages infected with *L. major* Seidman *wt* strains for 24 h, and treated with L-, RI- and D-BMAP-28 at 0.5 µM for an additional 24 h. Infected macrophages were stained with DAPI and examined under UV light with an upright fluorescent microscope, using 100 magnification. (B) Peritoneal macrophages were infection with *L. major* Seidman *wt* (filled) *and ko* strains (open) for 24 h, and treated with L-, RI- and D-BMAP-28 at 0.5 and/or 2 µM for 48 h. Infections were stained with DAPI and parasite burden was quantified as an average of 100 macrophages, and expressed as a percentage of the control infection. The average number of three complete biological replicates as well as the standard errors are shown. Paired T-tests untreated vs treated indicated significance, where * p<0.05, **p<0.005, ***p<0.0005.

In the course of these studies the BMAP-28 peptides demonstrated anti-parasitic activity against the promastigote stage of the parasite, however, activity against the morphological and biochemically differentiated amastigote form has not been shown. As the amastigote stage is the disease-causing stage of the parasite, it is essential for further therapeutic development that the peptides are also active against the amastigote stage. To assess the anti-amastigote activity of the peptides an assay was created where peritoneal-derived mouse macrophages were infected with the *L. major wt*, *ko and ko+* strains and subsequently treated with the BMAP-28 peptides at 0.5 and 2 µM concentrations. Following treatment with 0.5 µM peptide, *L. major wt* amastigote burdens in the infected macrophages were inhibited by 35%, 34% and 32% for L-, D- and RI-BMAP-28, respectively. Treatment with 2 µM concentrations of peptides further reduced amastigote burden by 82%, 80% and 67% for L-, RI- and D-BMAP-28, respectively. These data demonstrated that the amastigotes were killed upon the addition of the BMAP-28 isomers in a concentration dependent manner (Figure 6a and b).

Subsequently, this experiment was repeated using the *L. major* leishmanolysin *ko* strain to infect the macrophages, to investigate whether the absence of the leishmanolysin factor would play a role in the HDP effectiveness. As GP63 is down-regulated substantially in amastigotes, no significant differences were expected with regards to their anti-leishmania activity between the L-, RI- and D- forms of the peptide. As expected, there were no significant differences in killing activity between the L- form and the protease resistant RI- form of the peptide (Figure 6a and b). However, the D- form of the peptide was shown to be most effective at reducing the number of *L. major ko* amastigotes upon treatment with 0.5 µM D-BMAP-28. This differs dramatically from the *wt* infection where treatment of 0.5 µM D-BMAP-28 resulted in only 30% inhibition, demonstrating that the D-isoform of BMAP-28 was again more effective against the parasite.

## Discussion

*Leishmania* presents a prevalent protozoan parasite causing serious epidemics in the absence of adequate controls measures, and ineffective and outdated drug treatments. Novel leishmanicidal treatments are urgently required to combat this parasite. Ideally, these therapeutics would be pleotropic in their functions with both direct and selective leishmanicidal effects against the intracellular amastigote forms, as well as the ability to modulate the immune response of the host [34].

The potential of host defence peptides (HDPs) as novel anti-infective therapeutics has been derived from their role in nature to combat infectious agents within all living organisms through direct and/or indirect activities. Previously, several amphibian HDPs including magainin, temporin, dermaseptin, bombinin, and cecropin-melittin hybrid peptides have been shown to kill *Leishmania* by disruption of cell surface membranes resulting in osmotic lysis of the parasites [22], [35], [36], [37], [38], [39]. Similarly, the HDP histatin-5 (Hst5) has been shown to target the mitochondria of fungi and *Leishmania* [12]. Indeed, other mammalian cathelicidins such as protegrin-1 and BMAP-27 possess significant anti-parasitic activities (including *Leishmania*) [19], [20], thus it appears that these activities are conserved amongst all members of the cathelicidin family. The demonstration that many HDPs, including the cathelicidins, also function through modulation of host immune responses [13], [14], [15], [16], [17], [18] further supports their potential as anti-infective therapeutics including anti-parasitic pharmaceuticals.

BMAP-28, a 28 residue bovine cathelicidin HDP, has broad spectrum antimicrobial activities that include antibiotic-resistant bacterial clinical isolates and fungal pathogens [25] and induces membrane permeabilization and cell death in human tumor cell lines [25]. BMAP-28 also confers protection in various murine bacterial challenge models [23], [40] and reduces lethality to staphylococcal sepsis *in vivo* [24], [41].

As concerns have been raised regarding the therapeutic potential of HDPs due to their stability *in vivo* [42], strategies for improving HDP stability have been investigated primarily through the substitution of D-amino acids at the chiral centers of the HDPs [26], [36]. Similarly, modification of HDPs through retro-inversion by reversal of the peptide sequence and incorporation of D-amino acids also represents an intriguing strategy for increasing the therapeutic efficacy of HDPs [26]. Thus a comparative investigation of BMAP-28, D-BMAP-28 and RI-BMAP-28 appeared as a logical choice for the investigation protease resistant HDP anti-leishmanial therapeutics.

As anticipated from previous studies of other cathelicidins [19], [20], [21], [22], BMAP-28 has leishmanicidal activity against the promastigote *L. major* Friedlin and the Seidman strains as demonstated through a MTS assay of Leishmanial cell viability. Importantly our results demonstrate that D- and RI-BMAP-28 were also effective antimicrobials against *L. major* Friedlin and Seidman wild-type in a dose dependent manner. Interestingly, D-BMAP-28 was the most effective direct antimicrobial against both strains. This is perhaps less surprising as previous investigations with enantiomeric antimicrobial peptides have demonstrated equivalent or enhanced antimicrobial activities against both Gram-positive and Gram-negative bacteria as compared to their L-amino acid analogs [43], [44], [45]. RI-BMAP-28, which has virtually identical protease resistance profiles to D-BMAP-28 and similar seconday structure to L-BMAP-28 [26], also had improved leishmanicidal activity against the *L. major* Friedlin strain as compared to L-BMAP-28.

As a single therapeutic with immunomodulatory and leishmanicidal activites would be highly advantageous in the clinic we sought to verify the immunomodulatory activities of the three BMAP-28 isomers (Text S1). In addition to the chemotactic activities previously demonstrated for BMAP-28 [24], [25], [40], [46] and its RI- and all D- isomers [26], all three peptides were able to modulate chemokine secretion and inhibit LPS-mediated pro-inflammatory responses. An investigation by Santamaria *et al.* has also demonstrated the immunomodulatory potential of protease resistant peptides as enantiomeric pEM-2, an all-D amino acid derivative of a snake venom Lys49 phospholipase A_2_, significantly reduced LPS-induced TNF-α secretion from murine macrophages [44]. Thus, modification of HDPs by either inversion or retro-inversion appears to be a logical methodology for extending the biological half-lives of HDPs without undermining either leishmanicidal or immunodulatory activities of the peptide.

It has been demonstrated that the *Leishmania* surface metalloprotease leishmanolysin confers protection to the organism from the cytolytic properties of HDPs [20]. Leishmanolysin is the *Leishmania* surface-localized, zinc-dependent metalloproteinase and virulence factor, also known as GP63 or MSP [47], [48], [49]. Characterization of leishmanolysin has revealed that it is a neutral site-specific endopeptidase which preferentially cleaves peptides on the amino side of the recognition residue [50] and has a broad range of substrates [50], [51], [52], [53], [54]. Previously, a *L. major GP63* knockout mutant, *L. major ko*, was developed in which all 7 copies of GP63 were deleted [30]. As anticipated, the leishmanicidal activities of RI- and D-BMAP-28 were independent of GP63 whereas L-BMAP-28 was less effective in the presence of the leishmanolysin factor and more effective in the *ko* strain. These results supported previous findings that the presence of leishmanolysin protects the parasites from the cytolytic effects of various antimicrobial peptides through degradation [20]. Specifically, leishmanolysin has been shown to degrade the peptide pexiganan in the presence of zinc within a 15 min period [20]. Both *L. major wt* and *ko+* strains maintain these activities, which are absent from *L. major ko* strain [20].

It is interesting that at a concentration of 0.5 µM D-BMAP-28 was the most effective antimicrobial against the *ko* mutant whereas at 2 µM L-BMAP-28 was clearly the best peptide. As the antimicrobial activities of L-BMAP-28 are retained or improved following incorporation of D-amino acids it would be assumed that these activities represent non-chiral mechanisms of action. Thus, this may represent differences in the abilities of L- and D-BMAP-28 to associate and/or form active complexes in a concentration-dependent manner.

Differences were observed in the effectiveness of the peptides between the *L. major* Friedlin and Seidman strains as the peptides were more effective in the *L. major* Seidman strain than the Friedlin strain at equimolar concentrations. Although this emphasizes the importance of repeating such studies in several strains, the present work clearly demonstrates that protease resistant HDPs appear to have vastly improved leishmanicidal activities in comparison to their L- amino acid analogs.

Members of the cathelicidin family appear to exert their leishmanicidal activities through apoptotic and non-apoptotic mechanism of action resulting in significant membrane disruption, osmotic lysis and subsequent cell death [22], [25]. Our electron microscopy results demonstrated that all three BMAP-28 isomers caused significant disruption of membrane integrity in *L. major wt* cells similar to previous investigations of Hst5 [12] and bombinins H2/H4 [36]. Ultimately resulting in osmotic instability, vacuolar swelling, loss of cytosolic contents and eventual cell death. Protegrin, was previous shown to induce a nonapoptotic cell death in *L. major*, classified as annexin V negative, caspase 3/7 negative, Tunel assay negative and triggered a slow and steady response upon SYTOX incubation [20], [21]. Pexiganan, on the other hand induces an apoptotic cell death in *Leishmania* classified as annexin V positive, caspase 3/7 positive, Tunel assay positive and triggers a sustained response upon SYTOX incubation [20], [21]. Therefore, we postulated that the various BMAP-28 isomers would act through either pathway.

We utilised a SYTOX assay, a caspase 3/7 assay and a Tunel assay to classify the BMAP-28 induced cell death. The SYTOX assay results indicated a rapid and sustained response which would suggest an induction of an apoptotic response similar to that induced by pexiganan [21]. We subsequently investigated the caspase 3/7 activity and DNA laddering activity that resulted in response to D-BMAP-28 incubation, the most effective of the BMAP-28 peptides. There were minimal levels of caspase 3/7 activity in response to incubation with D-BMAP-28. Similarly there was no indication of DNA laddering in the stages of this induced response. This suggested a caspase-independent cell death. It was not until we investigated the induced late stage response to D-BMAP-28 that we were able to detect signs of apoptosis. At 24 hr it was clear that the D-BMAP-28 peptide induced apoptosis in all three cell strains. These results differ from those recently reported for pexiganan, where a caspase dependent apoptotic event was triggered early in response to peptide incubation [21], instead a late apoptotic event was triggered in response to incubation with D-BMAP-28.

Although the results from the *L. major* promastigote assays would suggest that BMAP-28 isoforms present a promising Leishmanial therapeutic candidate, this must be tempered with the understanding that it is the amastigote form of the parasite that causes disease in mammals. Upon infection promastigotes invade the macrophage cell where differentiate into amastigotes and take up residence in the lysosomal vacuole of the cell. Importantly, the amastigote differs dramatically both morphologically and metabolically from the promastigote form.

Previous studies involving murine *Brucella abotus* infection models have shown that antimicrobial peptides are able to enter the murine macrophage cell, evading lysis by macrophage enzymes and induce peptide mediated killing within a 24 hour period [55]. Thus we created a macrophage infection assay that mimicked mammalian infection to assess the activity of the BMAP-28 peptides on the amastigote parasite. Macrophages infected with *wt* parasites responded in a dose-dependent manner to treatment with the BMAP-28 peptides. When repeated using *ko* parasites to infect the macrophages, there were no major differences between the activity of the L- form of the peptide between *wt* and *ko* infections. As the leishmanolysin factor is significantly downregulated in the amastigote form of the parasite [56], leishmanolysin would be unable to impede the activity of the L- form of the peptide to the same extent as in the case of promastigotes. As anticipated, the D-form of the peptide, which was also shown to be effective at clearance of murine *Escherichia coli* infections upon prophylactic treatment [26], was more effective against the *L. major ko* amastigotes as compared to the *wt* amastigotes at the same concentration. The D- isoform was also most effective at reducing *L. major wt* promastigote cell viability.

Apart from its proteolytic role, leishmanolysin also has a number of other roles within the macrophage infection model that may explain the differences in activities amongst the three peptides between the wild-type and the *ko* amastigote strains. Leishmanolysin is involved in the direct interaction of promastigotes and the macrophage receptors where it serves as a ligand for binding macrophage complement [57], [58], as well as generating an additional epitope (C3bi) for binding to complement type 3 receptors [59]. Additionally, leishmanolysin *ko* mutants have increased complement sensitivity and less lesion development than *wt* parasites in mouse models of infection [30], [60].

Currently the majority of research into novel anti-leishmanial compounds and peptides focuses on their activities against the promastigotes form of the parasite. Our results clearly demonstrate the differences in activities of L-BMAP-28, D-BMAP-28 and RI-BMAP-28 between promastigotes and amastigotes and address the role of the leishmanolysin factor in reducing the activity of protease-susceptible compounds in promastigotes, but not in amastigotes. BMAP-28 and its isomers, D-BMAP-28 and RI-BMAP-28, are effective leishmanicidal compounds against promastigotes (insect infective form) *in vitro* inducing cell membrane disruption culminating in a late stage apoptotic cell death response. Moreover the BMAP-28 peptides were also effective at reducing amastigote cell viability within a macrophage infection model. Importantly, our results demonstrate that BMAP-28 is a promising template for the development of anti-infective therapeutics targeting *Leishmania*. It is imperative however that such design and development strategies address compound activity against both the promastigote and the amastigote form of the parasite.

## Supporting Information

Figure S1 **Inhibition of LPS-induced TNF-** ***a*** **secretion from human PBMCs by L-, D- and RI-BMAP-28.** PBMCs were incubated with 1.5 µM of L-BMAP 28, RI-BMAP-28, D-BMAP-28 or LL-37 (positive control) for 45 minutes before treating with highly purified *Pseudomonas aeruginosa* lipopolysaccharide (10 ng/ml). After 4 hours, TNF-α was measured in the supernatants by ELISA. Black bar: PBMC incubated with LPS alone. White bars: PBMC incubated with LL-37, L-BMAP 28, D-BMAP 28 or RI-BMAP 28. The average of three complete biological replicates are shown with standard errors.
(TIF)

Figure S2 **The effect of BMAP-28 peptides on** ***L. major*** **cells.** Wild-type cells were (A) untreated or treated with (B) L-BMAP-28, (C) RI-BMAP-28 or (D) D-BMAP-28 at 0.5 µM concentrations for 4 hours prior to fixation for TEM analyses.
(TIF)

Table S1 **The effect of BMAP-28 peptides on murine derived macrophages.** THP-1 cells were treated with 1 or 5 µM concentrations of L-, Ri or D-BMAP-28 for 24 or 72 hours. A trypan blue assay assessed the resulting cell viability and was recorded as a percentage. D- and RI-BMAP-28 retain immunomodulatory activities of L-BMAP-28. Although it was demonstrated that the D- and RI-BMAP-28 have retained or improved leishmanicidal activity of the parent peptide we also sought to investigate whether BMAP-28 and its protease resistant isomers may have the ability to modulate host immune responses including TNF-α mediated inflammatory responses. The D- and RI-BMAP-28 isomers retained the ability to induce the release of the chemokine MCP-1 in human PBMCs in a concentration-dependent manner similar to L-BMAP-28 (data not shown). All three peptides were also tested for their ability to inhibit LPS-induced TNF-α secretion in PBMCs. In three separate experiments, however, all of the BMAP-28 isomers strongly inhibited the induction of TNF-α secretion by LPS to the same degree as the human cathelicidin LL-37 (Figure S1). None of the BMAP-28 isomers directly induced TNF-α secretion.
(PDF)

Text S1 **D- and RI-BMAP-28 retain immunomodulatory activities of L-BMAP-28.**
(DOC)
