# Supplementary figures and images for: Effect of BMAP-28 Antimicrobial Peptides on Leishmania major Promastigote and Amastigote Growth: Role of Leishmanolysin in Parasite Survival
Source: PLoS Negl Trop Dis. 2011 May 31;5(5):e1141. doi: 10.1371/journal.pntd.0001141 (PMC3104953; doi:10.1371/journal.pntd.0001141)

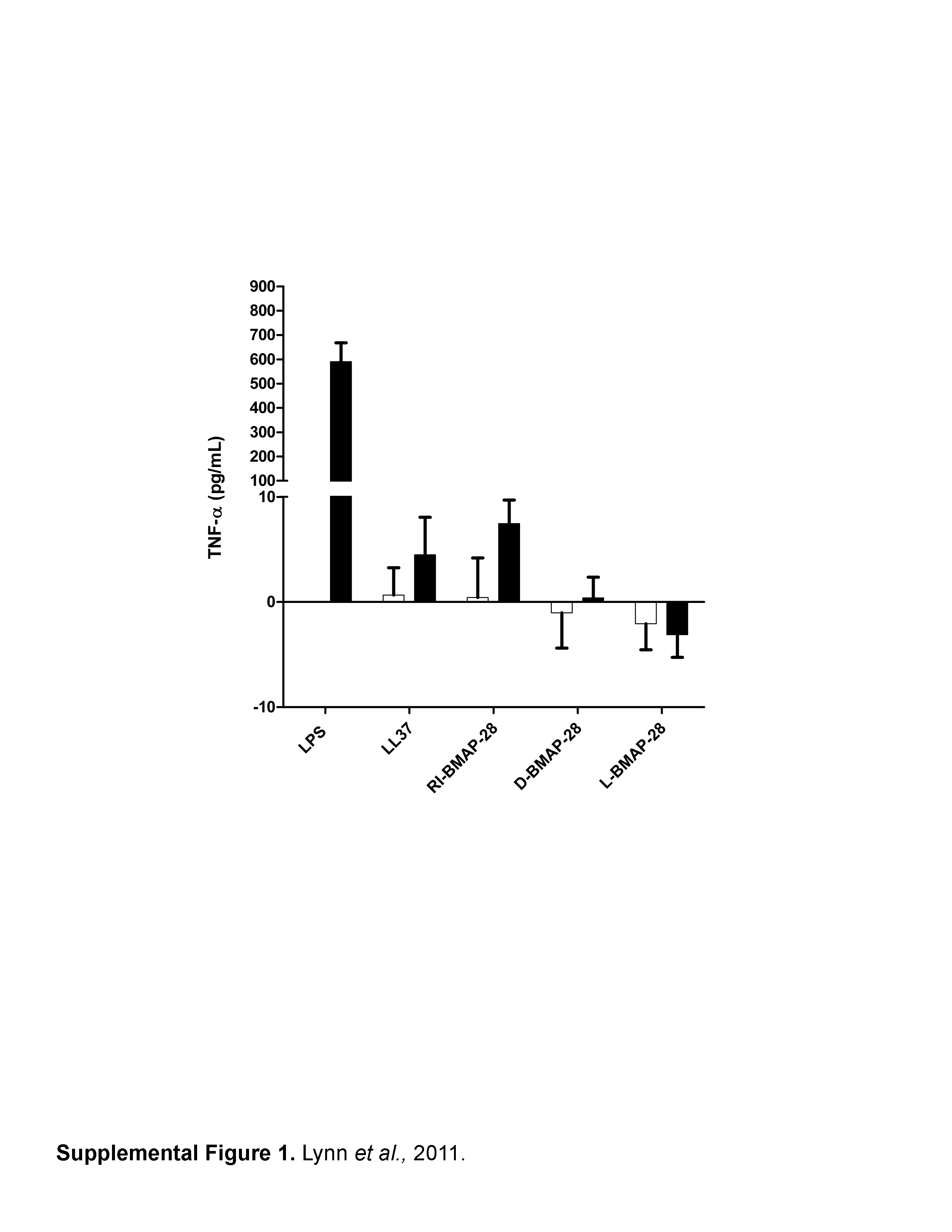

Supplement: Figure S1 — Inhibition of LPS-induced TNF- a secretion from human PBMCs by L-, D- and RI-BMAP-28. PBMCs were incubated with 1.5 µM of L-BMAP 28, RI-BMAP-28, D-BMAP-28 or LL-37 (positive control) for 45 minutes before treating with highly purified Pseudomonas aeruginosa lipopolysaccharide (10 ng/ml). After 4 hours, TNF-α was measured in the supernatants by ELISA. Black bar: PBMC incubated with LPS alone. White bars: PBMC incubated with LL-37, L-BMAP 28, D-BMAP 28 or RI-BMAP 28. The average of three complete biological replicates are shown with standard errors. (TIF) [file pntd.0001141.s001.tif]

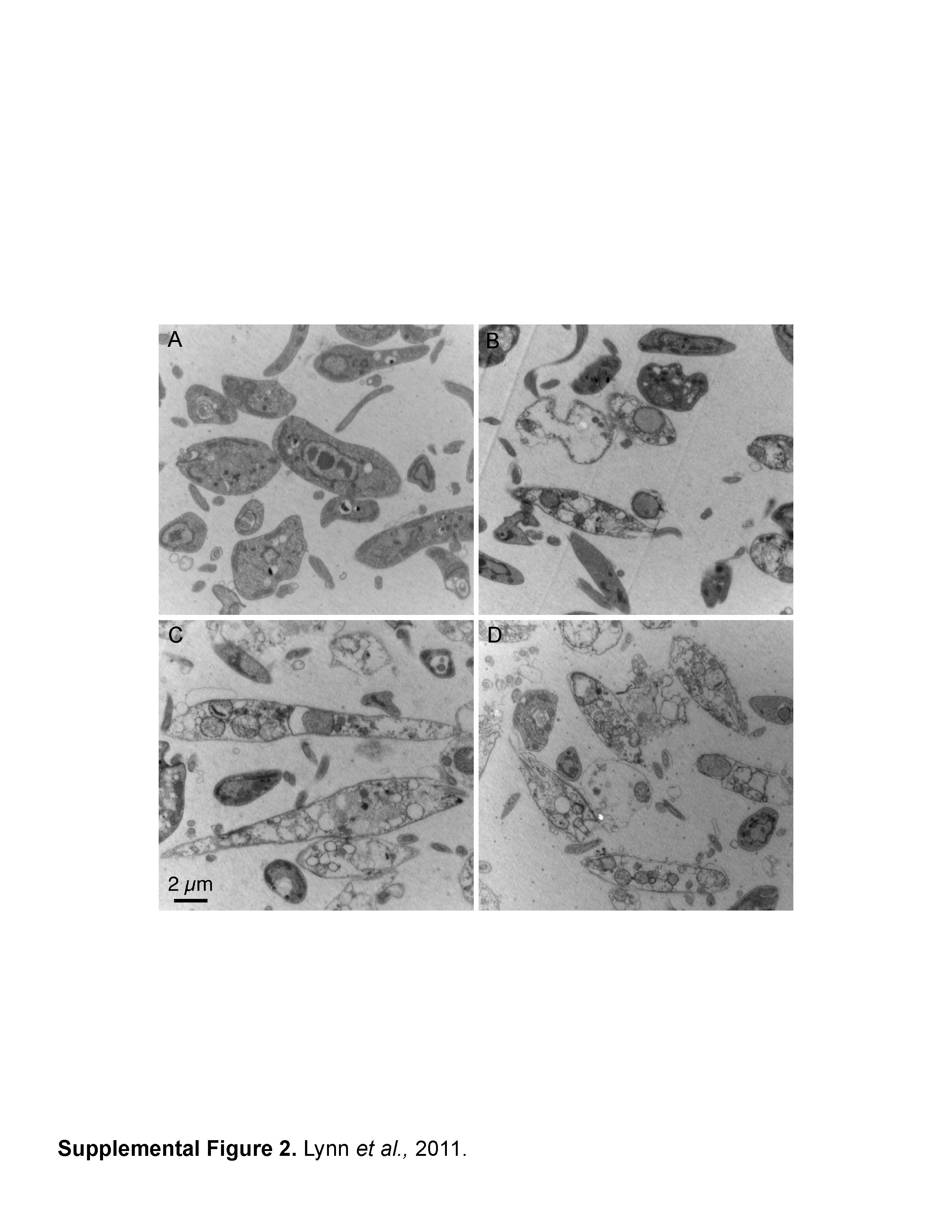

Supplement: Figure S2 — The effect of BMAP-28 peptides on L. major cells. Wild-type cells were (A) untreated or treated with (B) L-BMAP-28, (C) RI-BMAP-28 or (D) D-BMAP-28 at 0.5 µM concentrations for 4 hours prior to fixation for TEM analyses. (TIF) [file pntd.0001141.s002.tif]
