# Supplementary material for: Effect of BMAP-28 Antimicrobial Peptides on Leishmania major Promastigote and Amastigote Growth: Role of Leishmanolysin in Parasite Survival
Source: PLoS Negl Trop Dis. 2011 May 31;5(5):e1141. doi: 10.1371/journal.pntd.0001141 (PMC3104953; doi:10.1371/journal.pntd.0001141)
Supplement: Table S1 — The effect of BMAP-28 peptides on murine derived macrophages. THP-1 cells were treated with 1 or 5 µM concentrations of L-, Ri or D-BMAP-28 for 24 or 72 hours. A trypan blue assay assessed the resulting cell viability and was recorded as a percentage. D- and RI-BMAP-28 retain immunomodulatory activities of L-BMAP-28. Although it was demonstrated that the D- and RI-BMAP-28 have retained or improved leishmanicidal activity of the parent peptide we also sought to investigate whether BMAP-28 and its protease resistant isomers may have the ability to modulate host immune responses including TNF-α mediated inflammatory responses. The D- and RI-BMAP-28 isomers retained the ability to induce the release of the chemokine MCP-1 in human PBMCs in a concentration-dependent manner similar to L-BMAP-28 (data not shown). All three peptides were also tested for their ability to inhibit LPS-induced TNF-α secretion in PBMCs. In three separate experiments, however, all of the BMAP-28 isomers strongly inhibited the induction of TNF-α secretion by LPS to the same degree as the human cathelicidin LL-37 (Figure S1). None of the BMAP-28 isomers directly induced TNF-α secretion. (PDF) [file pntd.0001141.s003.pdf]

	Percentage cell viability	
	**24 h**	**72 h**
**1 µM L-BMAP**	**98.3**	**64.7**
**5 µM L-BMAP**	**96.4**	**68.7**
**1 µM Ri-BMAP**	**98.8**	**86.6**
**5 µM Ri -BMAP**	**96.5**	**87.7**
**1 µM D-BMAP**	**100**	**62**
**5 µM D-BMAP**	**94.9**	**55**
