# Supplementary material for: Effect of BMAP-28 Antimicrobial Peptides on Leishmania major Promastigote and Amastigote Growth: Role of Leishmanolysin in Parasite Survival
Source: PLoS Negl Trop Dis. 2011 May 31;5(5):e1141. doi: 10.1371/journal.pntd.0001141 (PMC3104953; doi:10.1371/journal.pntd.0001141)
Supplement: Text S1 — D- and RI-BMAP-28 retain immunomodulatory activities of L-BMAP-28. (DOC) [file pntd.0001141.s004.doc]

**Text S1**

Although it was demonstrated that the D- and RI-BMAP-28 have retained or improved leishmanicidal activity of the parent peptide we also sought to investigate whether BMAP-28 and its protease resistant isomers may have the ability to modulate host immune responses including TNF-α mediated inflammatory responses. The D- and RI-BMAP-28 isomers retained the ability to induce the release of the chemokine MCP-1 in human PBMCs in a concentration-dependent manner similar to L-BMAP-28 (data not shown). All three peptides were also tested for their ability to inhibit LPS-induced TNF-α secretion in PBMCs. In three separate experiments, however, all of the BMAP-28 isomers strongly inhibited the induction of TNF-α secretion by LPS to the same degree as the human cathelicidin LL-37 (Figure S1). None of the BMAP-28 isomers directly induced TNF-α secretion.
